# Distrust As a Disease Avoidance Strategy: Individual Differences in Disgust Sensitivity Regulate Generalized Social Trust

**DOI:** 10.3389/fpsyg.2016.01038

**Published:** 2016-07-28

**Authors:** Lene Aarøe, Mathias Osmundsen, Michael Bang Petersen

**Affiliations:** Political Science, Aarhus UniversityAarhus C, Denmark

**Keywords:** pathogen avoidance motivation, disgust sensitivity, trust, evolution, outgroup prejudice, generalized social trust, behavioral immune system, ideology

## Abstract

Throughout human evolutionary history, cooperative contact with others has been fundamental for human survival. At the same time, social contact has been a source of threats. In this article, we focus on one particular viable threat, communicable disease, and investigate how motivations to avoid pathogens influence people's propensity to interact and cooperate with others, as measured by individual differences in generalized social trust. While extant studies on pathogen avoidance have argued that such motivations should prompt people to avoid interactions with outgroups specifically, we argue that these motivations should prompt people to avoid others more broadly. Empirically, we utilize two convenience samples and a large nationally representative sample of US citizens to demonstrate the existence of a robust and replicable effect of individual differences in pathogen disgust sensitivity on generalized social trust. We furthermore compare the effects of pathogen disgust sensitivity on generalized social trust and outgroup prejudice and explore whether generalized social trust to some extent constitutes a pathway between pathogen avoidance motivations and prejudice.

## Introduction

One of the deadliest threats that humans have faced over the course of the evolutionary history of the species is the threat from pathogens. Defending against pathogens has shaped human physiology in multiple ways, one of the most obvious being the existence of the immune system. Importantly, however, research in psychology has recently demonstrated that the need to avoid pathogens has not only shaped human physiology but also human psychology (e.g., Schaller and Park, 2011; Schaller and Neuberg, 2012; Tybur et al., 2013). More specifically, it has been argued that the human immune system has a psychological or behavioral component, designed to demotivate people from engaging in behavior that would increase the likelihood of infections (e.g., Schaller, 2006). This so-called “behavioral immune system” works, at least in part, through the emotion of disgust and elicits disgust in the face of potential pathogenic contaminators, such as feces and rotten food (e.g., Schaller and Park, 2011; Schaller and Neuberg, 2012; Tybur et al., 2013).

For humans, one particularly powerful pathogen vector is other humans. Conspecifics are the species of larger size with which we interact the most and most intimately; pathogens that infect fellow humans are by definition dangerous to the human self; and many pathogens have a number of dedicated adaptations specifically designed for human-to-human transmission. While contact with other humans provides opportunities for increased fitness through social plus-sum games such as cooperation, other humans constitute at the same time a fundamental danger from a pathogen avoidance perspective. Hence, when trade-offs are made about whether to engage with others, pathogen avoidance motivations should motivate people to engage less rather than more. In this article, we therefore investigate the effects of pathogen avoidance motivations on one of the broadest and most fundamental measures of social perception: generalized social trust.

Generalized social trust is the willingness to trust others—including those we do not know (Putnam, 2000). It is a psychological heuristic that “provides a solution to the problems caused by social uncertainty” (Yamagishi and Yamagishi, 1994, p. 131; see also Petersen and Aarøe, 2015). More specifically, generalized social trust can be defined as “the belief that others will not deliberately or knowingly do us harm, if they can avoid it, and will look after our interests, if this is possible” (Delhey and Newton, 2005, p. 311). Generalized social trust is a stable individual difference that influences social behavior in a range of contexts, including collective action and cooperation (e.g., Uslaner, 2002), the provision of public goods (Sønderskov, 2009), and investments in local communities (Putnam, 2000). Importantly, generalized social trust promotes social contact of a particular type. It is different from trust in individuals with whom we are familiar, such as family (Uslaner, 2002). Instead, generalized social trust tracks trust and engagement in “weak social ties”—that is, the people within an individuals' extended social network—and is typically linked in modern societies to membership of and engagement in associations, clubs, and communities (Putnam, 2000; Uslaner, 2002; see also Granovetter, 1973 for further discussion of the concept of weak social ties).

Here, we focus on whether individual differences in generalized social trust reflect individual differences in the activation of the behavioral immune system. Hence, due to a combination of situational, developmental, and genetic factors, people differ in their degree of activation of the behavioral immune system and the resulting feelings of disgust. Some people are simply more “disgust sensitive” (e.g., Schaller and Park, 2011; Schaller and Neuberg, 2012; Tybur et al., 2013). Are they also more likely to avoid engaging with others as reflected in a lower level of generalized social trust?

While past research has investigated the effects of individual differences in pathogen avoidance motivations on social perceptions, we part in a fundamental way with the focus of this past research. It has previously been demonstrated that higher levels of pathogen avoidance motivations predict more negative perceptions of groups of people that can be construed as outgroups, such as foreigners, immigrants, and gays (e.g., Faulkner et al., 2004; Park et al., 2007; Green et al., 2010), and it has been argued that pathogen avoidance motivation is one of the core individual differences underlying social conservatism (Terrizzi et al., 2013; although see Tybur et al., 2015a,b). In contrast, we argue, if individuals are guided by pathogen avoidance motivations, it should not be people specifically from outgroups that are tagged as dangerous. It should be people more broadly (including ingroup members) as, for example, reflected in lower levels of generalized social trust.

The argument in the existing literature that outgroups are a key target of pathogen avoidance motivations builds on the notion of immunization and how immunization toward specific pathogens is acquired through exposure to them (as utilized in vaccination programs). If individuals from other groups have a different pathogen ecology than the self's group, this means that inter-group contact would expose the self to pathogens for which the self may have no immunity. This is potentially lethal, as demonstrated by the Spanish conquest of the Americas, where some 90% of the native population was killed by a smallpox epidemic brought overseas by the Spanish conquistadors (Diamond, 1997). Existing research has highlighted this prominent case (e.g., Faulkner et al., 2004) and, if such instances have been recurrent over human evolutionary history, the behavioral immune system might have evolved by natural selection in order to activate social avoidance motivations specifically toward people from other groups.

The problem is, however, that instances such as the conquest of the Americas are unlikely to have been recurrent with any high frequency over human evolutionary history. Firstly, the low ancestral migration rates—because our ancestors traveled by foot for the vast majority of evolutionary history—make the classical model an unlikely model of recurrent between-group contact (Kurzban et al., 2001). Secondly, while one study demonstrates variation in immunization within short distances in the Amazonas, as pointed out in the study, this is only found for modern, highly infectious diseases that most likely have evolved recently (Black, 1975). Third and finally, the argument that outgroup pathogens are particularly dangerous can be turned fully on its head: the adaptation of pathogens to local environments could imply that they are less lethal for people outside of that environment, including members of other groups (de Barra and Curtis, 2012).

Instead, we suggest that individual differences in pathogen avoidance motivations reflect self-oriented trade-offs about the pros and cons of contact with others such that those who are motivated by pathogen avoidance are less likely to seek out cooperative plus-sum games more broadly rather than specifically avoiding outgroup members. In modern society, cooperative plus-sum games can easily be established without any form of physical contact using information technology. Nevertheless, human pathogen avoidance psychology can be expected to have acquired its design under ancestral, evolutionarily recurrent conditions before the advent of modern technologies. To understand how this psychology exactly should shape social avoidance, we must therefore attend to the details of ancestral sociality (e.g., Kurzban and Leary, 2001; Faulkner et al., 2004; Navarrete and Fessler, 2006; Schaller, 2006).

Humans evolved as hunters and gatherers, typically operating in small-scale bands of about 30 individuals. According to the anthropological evidence, these forager bands most likely belonged to extended social networks, sometimes referred to as tribes, alliances, or meta-groups (Kelly, 1995; Hill et al., 2011; Walker et al., 2013). The nodes of these networks were all part of the same ingroup: goods, mates, and information were exchanged through the links of the network, and members of a single network engaged in joint competition, including warfare, against other similar networks (i.e., outgroups). The members of individual forager bands were drawn from this larger meta-group and their composition changed on a seasonal basis through fusion–fission processes (Kelly, 1995). To some extent, however, close kin (especially parents and offspring) would have tended to stay together and, hence, formed stable nucleuses within dynamically changing bands.

For ancestral types of crucial, within-band cooperation, it is difficult to avoid physical contact. Moreover, there is substantial archaeological and anthropological evidence that kin and band members play a key role in buffering against the consequences of sickness and injury by engaging in health care toward affected band members (Sugiyama, 2004; Jensen and Petersen, 2016). Hence, pathogen avoidance motivations will not likely influence social motivations toward close kin and friends. Ancestrally, however, individuals who were motivated to avoid pathogens could down-regulate engagement in the broader social network, for example by traveling less to other nodes in the network, and be less likely to engage in band fusions^1^. While the evidence on humans is limited, studies of social networks among giraffes show that such “weak social ties” are responsible for the spread of communicable diseases (VanderWaal et al., 2016).

Trust and engagement in “weak social ties” (Granovetter, 1973)—that is, people within an individual's extended social network—are what generalized social trust has been found to track (e.g., Putnam, 2000; Uslaner, 2002). For example, Uslaner (2002, pp. 53–54) finds that trust in “people where you shop,” “people you meet on the street,” “most people,” and trust in “neighbors” all reflect generalized trust. Hence, we predict that pathogen avoidance motivations will down-regulate generalized social trust.

There is one potential challenge to our argument: the observation of extensive individual differences in behavioral immune activation could undercut any use of the behavioral immune system as a protection mechanism. If just one family or band member is open to extensive contact with others, others within the band—even if personally less open—would also suffer. Hence, a premise of our argument is that ancestral variation in pathogen avoidance motivations within bands (or potentially within entire tribes) was less than what is observed within societies today. This is plausible because ecological conditions (e.g., objective pathogen exposure and other factors that could calibrate the activation of the behavioral immune system) would vary less ancestrally than today. Consistent with this, studies of the personalities of foragers have also shown them to be less varied than the personalities of individuals in modern, Western cultures (Gurven et al., 2013)^2^.

It is also important to note that our argument concerning the effect of pathogen avoidance motivations on generalized social trust does not necessarily entail that there are no additional avoidance motivations toward people who are considered true outgroup members. But we predict that wariness of people in general is a core output of the behavioral immune system and argue that this ought to be the first-order prediction about how pathogen avoidance motivations influence social cognition. To our knowledge, only one study has investigated a corollary hypothesis and identified a positive correlation among humans between individual differences in disgust sensitivity and the need for larger personal spaces in interpersonal interactions (Park, 2015). This interesting finding is aligned with the argument advanced here but leaves unexamined whether high pathogen avoidance motivation not only motivates people to avoid interactions with outgroups but prompts them to avoid others in general.

As we will demonstrate, our investigation of the influence of individual differences in pathogen avoidance motivations on generalized social trust not only provides important insights into the structure of the behavioral immune system but also regarding the nature of its effects on broader psychological constructs, such as political attitudes about outgroups and social conservative ideology.

## Study 1: does high pathogen avoidance motivation reduce social trust?

The aim of Study 1 is to investigate whether individual differences in pathogen avoidance motivations predict generalized social trust. If pathogen avoidance motivations indeed constitute a psychological motivation designed to not only motivate people to avoid interactions with outgroups but prompt them to avoid others in general, high pathogen avoidance motivation should reduce generalized social trust.

### Materials and measures

To provide a first test of this argument, we implemented a survey using Amazon Mechanical Turk (MTurk). The survey was completed by 508 respondents. Of these, 39% were female, and the average age was 35 years (*SD* = 11 years). In terms of education, 1% had not graduated from high school, 12% were high school graduates, 34% had some college or were currently enrolled in college, 41% were college graduates, and 12% had a post-college degree. The median family income of the respondents was “between $35,000 and $49,999”^3^.

As our measure of pathogen avoidance motivations, we rely on the 7-item pathogen disgust scale from the Three Domain Model of Disgust (Tybur et al., 2009). Pathogen disgust “is elicited by objects likely to contain infectious agents, including dead bodies, rotting foods, and bodily fluids such as feces, phlegm, vomit, blood, and semen, and it motivates proximal avoidance of such things” (Tybur et al., 2009, p. 105). The pathogen disgust scale has good reliability and is conceptually distinct from other disgust domains related to sexuality and morality (Tybur et al., 2009). To assess individual differences in pathogen disgust sensitivity, the participants rated how disgusting they would find each of the following concepts on 7-point scales: “Stepping on dog poop,” “Sitting next to someone who has red sores on their arm,” “Shaking hands with a stranger who has sweaty palms,” “Seeing some mold on old leftovers in your refrigerator,” “Standing close to a person who has body odor,” “Seeing a cockroach run across the floor,” and “Accidentally touching a person's bloody cut” (e.g., Tybur et al., 2009). The items were summed to a reliable scale (α = 0.82) ranging from 0 to 1, higher values indicating stronger pathogen disgust sensitivity (mean = 0.61, *SD* = 0.18, min. = 0.05, max. = 1).

As our measure of social trust we use the classical question developed by Rosenberg (1956) which has become the international standard single-item trust measure that is included in, for example, the European Social Survey, the European Values Study, World Values Survey, and General Social Survey (e.g., Uslaner, 2002, p. 52; Inglehart and Welzel, 2005, p. 22; Moreno, 2011, p. 2672; Petersen and Aarøe, 2015, p. 1683). Specifically, the respondents were asked to indicate: “Generally speaking, would you say that most people can be trusted or that you can't be too careful in dealing with people? Please indicate your answer on a score of 0–10, where 0 means you can't be too careful and 10 means that most people can be trusted.” Answers were measured on an 11-point scale with endpoints labeled “You can't be too careful” and “Most people can be trusted.” The answers were subsequently rescaled to range from 0 to 1, higher values indicating higher trust (mean = 0.51, *SD* = 0.25, min. = 0, max. = 1)^4^.

We also include a number of socio-demographic control variables. Specifically, we control for gender, age, education, family income, and race (1 = Caucasian, 0 = racial/ethnic minority groups; see the Online Appendix A1 “Supplemental measurement details for Study 1” for the full detail on the question wording and coding of socio-demographic control variables). Past research on individual level factors explaining social trust emphasizes that “[t]he most common predictors of trust are, of course, personal resource variables” (Stolle, 1998, p. 512), as reflected in, for example, income and education (e.g., Putnam, 1995; Verba et al., 1995). Likewise, prior research has also found that age influences social trust, meaning that older people tend to have higher social trust (Neller, 2008, p. 109). While some studies have also found an effect of gender, the results are mixed. We follow state of the art practice in the literature and control for both gender and age (Neller, 2008, p. 109). All variables are scaled from 0 to 1 except for age, which is measured in years.

We report unstandardized OLS regression coefficients for effect-size coefficients. Because of the scaling of the variables from 0 to 1, the OLS regression coefficients “can be interpreted as indicating the change in percentage points of the full dependent-measure scale when the independent measure changes from its lowest to its highest value” on the scale (Petersen and Aarøe, 2015, pp. 1683–1684). This is perceived to be the best effect-size measure for regression in econometrics (Achen, 1982, pp. 76–77). In all of the tests reported in the manuscript, the data were analyzed in Stata 14.

### Results

To assess the relationship between social trust and pathogen avoidance motivations, we regress generalized social trust on individual differences in pathogen disgust sensitivity and control variables. Specifically, Model 1 in Table 1 shows the bivariate association between individual differences in pathogen disgust sensitivity and generalized social trust. In Model 2 we add control for socio-demographic factors related to gender, age, education, and family income. Entries in Table 1 are unstandardized OLS regression coefficients with robust standard errors in parentheses^5^. All variables range between 0 and 1 except for age which is measured in years.

**Table 1:** **Social trust and individual differences in pathogen disgust sensitivity**.

	**M1**	**M2**
Pathogen disgust	−0.16^*^ (0.07)	−0.18^*^ (0.07)
Education		0.05 (0.05)
Income		0.14^**^ (0.05)
Caucasian		0.01 (0.02)
Female		0.03 (0.02)
Age		0.00 (0.00)
Constant	0.61^***^ (0.04)	0.47^***^ (0.07)
*n*	506	506
*R*^2^	0.014	0.047

*Entries are unstandardized OLS regression coefficients. Robust standard errors are reported in parentheses*.

* *p < 0.05*,

** *p < 0.01*,

*** *p < 0.001*.

The results in Table 1, Models 1–2, show a substantial and statistically significant association between individual differences in pathogen disgust sensitivity and generalized social trust. Individuals who are high in pathogen disgust sensitivity express significantly lower social trust than individuals low in pathogen disgust sensitivity (b_Model1_ = −0.16, *p*_Model1_ = 0.019, b_Model2_ = −0.18, *p*_Model2_ = 0.013). Importantly, this relationship replicates across the bivariate analysis (Model 1) and controlling for fundamental socio-demographic variables (Model 2).

Importantly, the findings in Table 1, Model 2, also highlight the substantive importance of the direct effect of individual differences in pathogen disgust on generalized social trust. The extent of the effect of individual differences in pathogen disgust sensitivity is three times as large as the direct effect of education, which is not even significant in the model. Likewise, the findings in Table 1, Model 2, also emphasize that the effect of pathogen disgust sensitivity is comparable to the direct effect of family income on social trust.

In summary, the findings in Study 1 support the prediction that individual differences in pathogen avoidance motivations shape generalized social trust. Likewise, the results also support that pathogen avoidance motivations regulate generalized social trust over and beyond traditional socio-demographics, including education, which past research has emphasized to be one of the most central factors explaining individual differences in generalized social trust.

## Study 2: increasing measurement validity, reliability, and internal validity

The findings in Study 1 consistently support that pathogen avoidance motivations predict trust in others in general as measured by the international standard single-item measure of generalized social trust. As emphasized by Freitag and Traunmüller (2009, p. 784), however, “[g]eneralized social trust is a rather abstract attitude toward people in general, encompassing those beyond immediate familiarity, including strangers.” Some research argues that the international standard single-item is underspecified, meaning that “respondents will have to fill in their own specifications” (Nannestad, 2008, p. 417, see also Freitag and Bauer, 2013, p. 29). Hence, the central aim of Study 2 is to rule out that the findings reported in Study 1 are driven by people who are thinking about outgroups when reporting their generalized social trust.

A second aim of Study 2 is to examine the robustness of the findings from Study 1 by introducing new control variables related to personality to increase internal validity. Regarding personality, prior studies have shown that personality traits, as indexed by the Big Five, correlate with individual differences in disgust sensitivity (e.g., Druschel and Sherman, 1999). Furthermore, personality traits are also associated with social trust (e.g., Mondak and Halperin, 2008; Dinesen et al., 2014). Controlling for Big Five traits ensures that any detected association between pathogen disgust sensitivity and social trust is not merely a reflection of the personality traits already identified in prior research (e.g., agreeableness, openness to experience, or neuroticism as the three main candidates related to trust and disgust sensitivity, respectively).

### Materials and measures

We implemented a survey using Amazon Mechanical Turk (MTurk), which was completed by 1422 respondents. The average age was 35 years (*SD* = 11 years), and 45% were female. In terms of education, 1% had not graduated from high school, 9% were high school graduates, 31% had some college or were currently enrolled in college, 43% were college graduates, and 15% had a post-college degree. The median family income was “between $35,000 and $49,999.”

To directly test that the findings in Study 1 do not reflect that people think about outgroups when indicating their trust in “most people,” we implemented a question wording experiment in the survey. The respondents were randomly assigned to one of two conditions. Specifically, we varied whether respondents were asked about their trust in (1) “most people,” (i.e., the standard formulation) or (2) “people in your neighborhood,” the latter referring to a specific ingroup of the “weak social ties”-type: people who live in the respondents' local area but whom they do not necessarily know. If, contrary to our argument, the effects of pathogen avoidance motivations on trust in “most people” are significantly driven by outgroup prejudice, then we should find significantly different effects of these motivations on trust across these two conditions. The use of an experiment in this regard provides strong causal leverage, as the random assignment to conditions ensures that the rating of one of the groups does not affect the rating of other groups.

**Figure 1:**
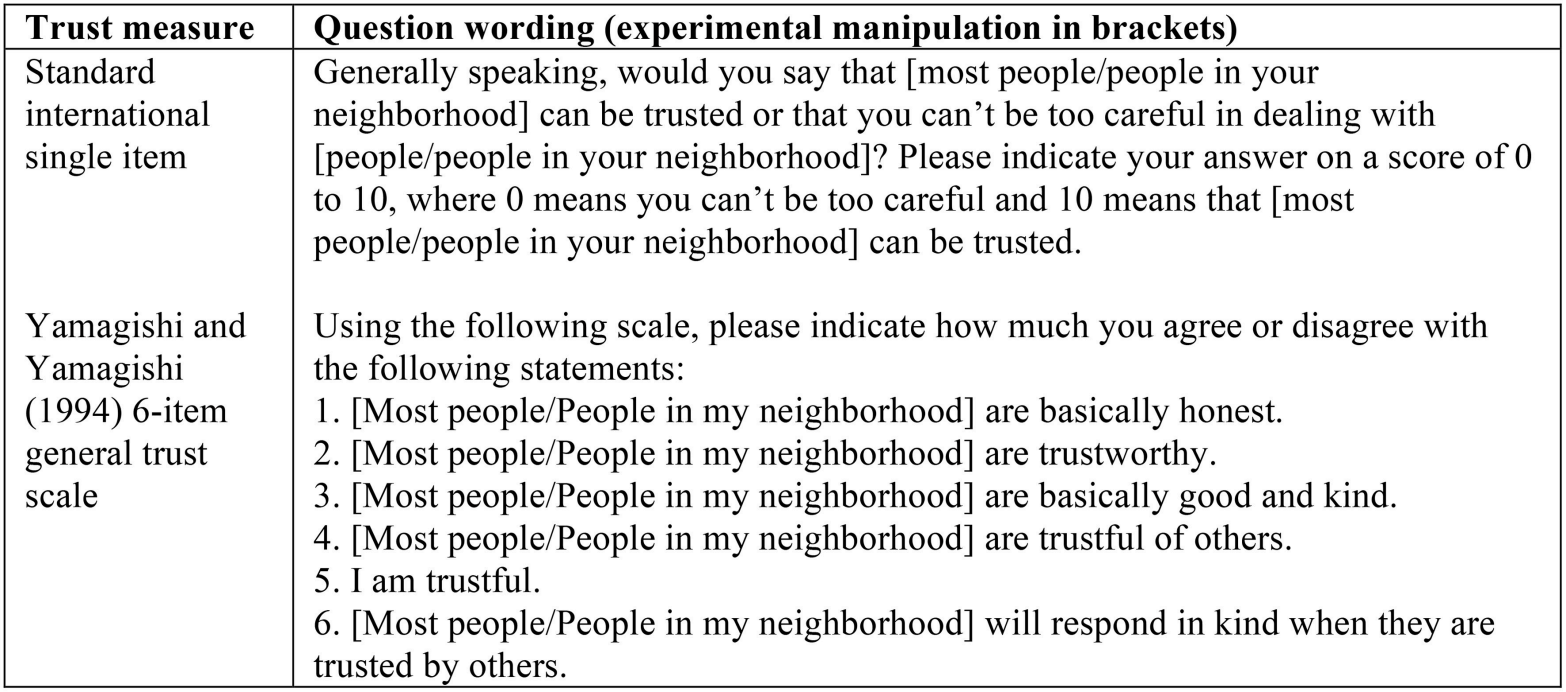
**Question wording with experimental manipulations in square brackets**.

We implemented this variation in the question wording in the international standard single-item trust question and in the Yamagishi and Yamagishi (1994) 6-item General Trust scale to increase reliability by adding a trust scale among our dependent variables. The specific question wording in the two experimental conditions is reported in Figure 1 below with the experimental variations in square brackets.

Answers to the single item were measured on a scale ranging from 0 to 10 with endpoints labeled “You can't be too careful” and “Most people can be trusted” in the “most people” condition, and “Can't be too careful in dealing with people in my neighborhood” and “People in my neighborhood can be trusted” in the “neighborhood” condition (mean = 0.56, *SD* = 0.26, min. = 0, max. = 1, across the two conditions on a scale ranging from 0 to 1, higher values indicating higher trust). This is our first trust measure in the experiment^6^.

Answers to the six items from the Yamagishi and Yamagishi (1994) General Trust scale were measured on 5-point scales with endpoints labeled “strongly disagree” and “strongly agree.” Answers were summed to a reliable scale and re-scaled to range from 0 to 1, higher values indicating higher trust (α = 0.89, mean = 0.64, *SD* = 0.18, min. = 0, max. = 1, across the two conditions). This is our second trust measure in the experiment^7^.

Finally, as our third trust measure we combine the Yamagishi and Yamagishi (1994) general trust scale and the standard single trust item into a combined generalized trust measure (*r* = 0.76, mean = 0.60, *SD* = 0.21, min. = 0, max. = 1 on a scale ranging from 0 to 1)^8^.

After the experiment, we asked subjects to complete a question battery from the World Value Study designed to measure trust in various groups across a “radius of trust from the most particular to the most general” including outgroups (Zmerli and Newton, 2013, p. 72). This provides us with a second, more exploratory possibility of testing how pathogen avoidance motivations shape social cognition. In the original question battery from the World Value Study, respondents are asked to indicate their trust in people from six various groups. To obtain a more fine-grained measure including a larger variety of in- and outgroups, we added three groups (marked with ^*^below): Specifically, the respondents were asked, “I'd like to ask you how much you trust people from various groups. Could you tell me for each whether you trust people from this group completely, somewhat, not very much or not at all?”: “1. Your family,” “2. Your friends”^*^, “3. Your neighborhood,” “4. People you know personally,” “5. People you meet for the first time,” “6. People of another religion,” “7. People of another nationality,” “8. People of another ethnicity”^*^, “9. Immigrants”^*^ (the groups appeared in the listed order). Answers were measured on a 4-point scale with endpoints labeled “Do not trust at all,” and “Trust completely.” All nine items were rescaled to range from 0 to 1, higher values indicating higher trust (see the Online Appendix Table A2 in “A3. Supplemental measurement details for Study 2” for mean, standard deviation, minimum and maximum on each item). As argued in the theory section, we do not expect pathogen avoidance motivations to predict trust in family members. At the same time, it is important for our argument that the effects of pathogen avoidance motivations extend beyond trust in outgroups, such as groups 6, 7, 8, and 9.

To measure pathogen avoidance motivations, we use the same measure as in Study 1, that is, the 7-item pathogen disgust scale from the Three Domain Model of Disgust (Tybur et al., 2009). Answers were summed to a satisfactorily reliable scale ranging from 0 to 1, higher values indicating higher pathogen disgust sensitivity (α = 0.82, mean = 0.62, *SD* = 0.18, min. = 0, max. = 1).

To measure individual differences in personality as indexed by the Big Five inventory, we rely on the validated 10-item instrument developed by Gosling et al. (2003). All answers were summed into satisfactorily reliable scales ranging from 0 to 1 (*r* = 0.35 or higher) (see the Online Appendix “A3. Supplemental measurement details for Study 2” for full question wordings and description of the scale constructions).

Finally, the socio-demographic control variables gender, age, education, family income, and race (1 = Caucasian, 0 = racial/ethnic minority groups) were measured and coded as in Study 1.

### Results

We begin by testing whether the effect of individual differences in pathogen disgust sensitivity on trust differs depending on whether people are asked about trust in “most people” or in “people from your neighborhood,” that is, a “weak social ties” ingroup. Most importantly, we observe no moderating effect of experimental condition on the effect of pathogen disgust sensitivity on trust [b_pathogen disgust × experimental condition_ = 0.06, *p* = 0.435 in the model for the single item trust measure; b_pathogen disgust × experimental condition_ = 0.04, *p* = 0.423 in the model for the Yamagishi and Yamagishi (1994) General Trust scale; and b _pathogen disgust × experimental condition_ = 0.05, *p* = 0.395 in the model for the combined generalized trust measure; see the Online Appendix A4.1 “Interaction analyses for Study 2” for the full interaction models]^9^. Consistent with our theoretical argument, the effect of pathogen disgust sensitivity on trust does not differ statistically depending on whether people are asked about “most people” or a genuine ingroup of the “weak social ties”-type. Hence, the effect of pathogen avoidance motivations on the standard measure of generalized social trust is not completely driven by outgroup prejudice.

**Figure 2:**
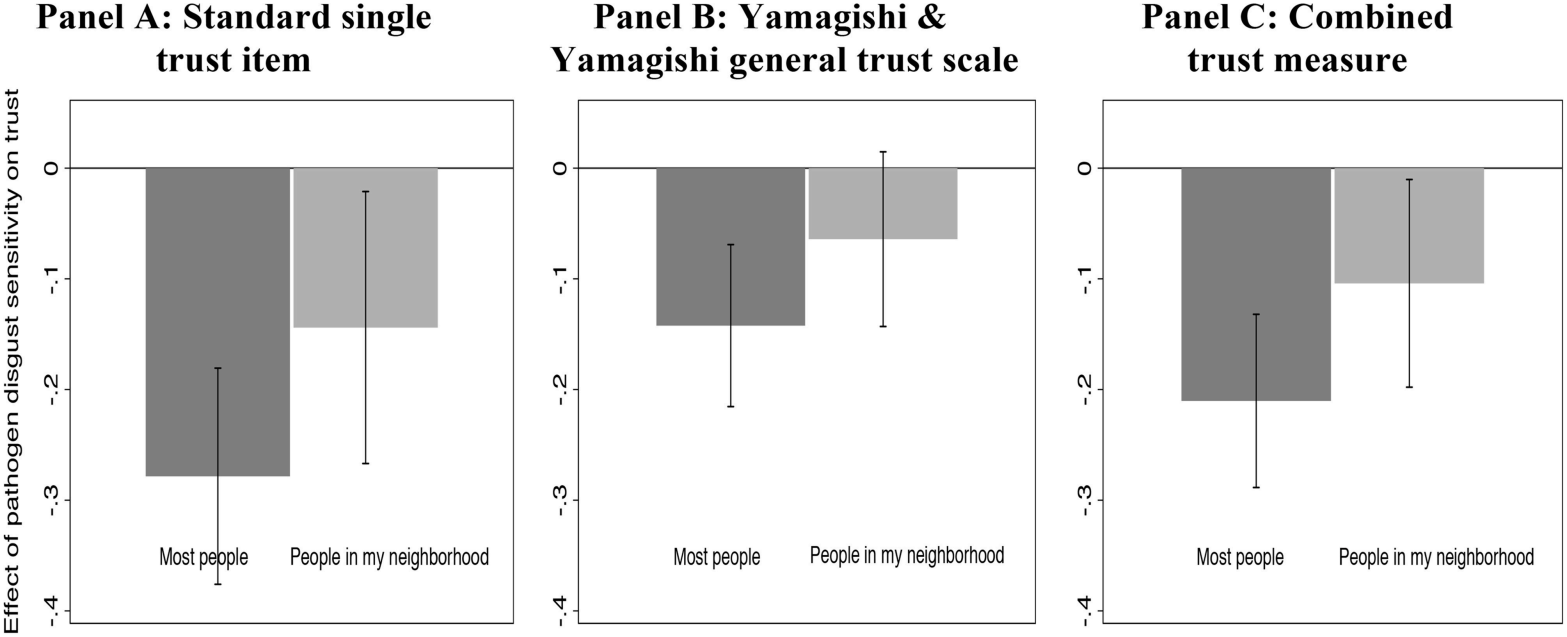
**Effect of pathogen disgust on trust by trust measure and experimental condition. (A)** Standard single trust item; **(B)** Yamagishi and Yamagishi general trust scale; **(C)** Combined trust measure. Note. Entries are unstandardized beta-coefficients for the effect of pathogen disgust sensitivity on trust by experimental condition and trust measure. Pathogen disgust sensitivity and the three trust measures were scaled from 0 to 1. The coefficients were estimated using OLS regression and controlling for the same variables as in Table 2, *n* = 711–709 in each model. The full regression results are reported in the Online Appendix Table A4.

By experimental condition and trust measure, Figures 2A–C display the effects of individual differences in pathogen disgust sensitivity on trust controlling for socio-demographic factors and personality as indexed by the Big Five. Entries are unstandardized OLS regression coefficients with 95% confidence intervals. Coefficients were estimated using OLS regression, and the models included the same control variables as in Table 2 (see below; the full regression models are reported in the Online Appendix Table A4)^10^.

**Table 2:** **Trust in people from various groups and individual differences in pathogen disgust sensitivity**.

	**Family**	**Friends**	**People you know personally**	**Your neighborhood**	**People you meet for the first time**	**People of another religion**	**People of another nationality**	**People of another ethnicity**	**Immigrants**
	**M1**	**M2**	**M3**	**M4**	**M5**	**M6**	**M7**	**M8**	**M9**
Pathogen disgust	−0.02 (0.03)	−0.06^†^ (0.03)	−0.06^†^ (0.03)	−0.08^*^ (0.04)	−0.20^***^ (0.04)	−0.17^***^ (0.04)	−0.17^***^ (0.04)	−0.17^***^ (0.04)	−0.23^***^ (0.04)
Education	0.07^*^ (0.03)	0.05^†^ (0.03)	0.03 (0.02)	0.06^*^ (0.03)	0.06^*^ (0.03)	0.02 (0.03)	0.05 (0.03)	0.04 (0.03)	0.07^*^ (0.03)
Income	0.13^***^ (0.02)	0.08^***^ (0.02)	0.05^*^ (0.02)	0.12^***^ (0.03)	0.07^**^ (0.03)	0.08^***^ (0.02)	0.05^*^ (0.02)	0.06^**^ (0.02)	0.04^†^ (0.02)
Caucasian	−0.00 (0.01)	0.04^**^ (0.01)	0.01 (0.01)	0.06^***^ (0.01)	0.06^***^ (0.01)	0.04^**^ (0.01)	0.01 (0.01)	0.00 (0.01)	0.00 (0.01)
Female	−0.00 (0.01)	0.01 (0.01)	0.03^**^ (0.01)	−0.00 (0.01)	0.01 (0.01)	0.03^**^ (0.01)	0.05^***^ (0.01)	0.05^***^ (0.01)	0.05^***^ (0.01)
Age	−0.00 (0.00)	−0.00 (0.00)	−0.00 (0.00)	0.00^**^ (0.00)	0.00 (0.00)	0.00 (0.00)	−0.00 (0.00)	0.00 (0.00)	0.00 (0.00)
Openness	−0.07^**^ (0.03)	−0.01 (0.03)	−0.05^†^ (0.03)	−0.05 (0.03)	−0.08^*^ (0.03)	0.01 (0.03)	0.04 (0.03)	0.07^*^ (0.03)	0.09^**^ (0.03)
Conscientiousness	0.02 (0.03)	0.00 (0.03)	0.04 (0.03)	0.05 (0.03)	−0.13^***^ (0.03)	−0.07^*^ (0.03)	−0.05 (0.03)	−0.06^†^ (0.03)	−0.08^*^ (0.03)
Extraversion	0.01 (0.02)	−0.00 (0.02)	0.04^*^ (0.02)	0.02 (0.02)	0.09^***^ (0.02)	0.03 (0.02)	0.02 (0.02)	0.03 (0.02)	−0.02 (0.02)
Agreeableness	0.19^***^ (0.03)	0.19^***^ (0.03)	0.13^***^ (0.03)	0.12^**^ (0.04)	0.24^***^ (0.04)	0.19^***^ (0.03)	0.18^***^ (0.03)	0.18^***^ (0.04)	0.15^***^ (0.04)
Neuroticism	−0.12^***^ (0.03)	−0.11^***^ (0.03)	−0.09^***^ (0.02)	−0.10^**^ (0.03)	−0.12^***^ (0.03)	−0.03 (0.03)	−0.03 (0.03)	−0.04 (0.03)	−0.06^*^ (0.03)
Constant	0.74^***^ (0.05)	0.65^***^ (0.05)	0.60^***^ (0.05)	0.37^***^ (0.05)	0.38^***^ (0.05)	0.44^***^ (0.05)	0.47^***^ (0.05)	0.45^***^ (0.05)	0.49^***^ (0.05)
*n*	1420	1420	1420	1420	1420	1420	1420	1420	1420
*R*^2^	0.116	0.106	0.085	0.112	0.132	0.091	0.080	0.090	0.093

*Entries are unstandardized OLS regression coefficients. Robust standard errors are reported in parentheses*.

† p < 0.64

* *p < 0.05*,

** *p < 0.01*,

*** *p < 0.001*.

As seen in Figure 2A, we observe a significant association between pathogen disgust sensitivity and generalized social trust as measured using the single item no matter whether the question wording targets “most people” (*b* = −0.28, *p* < 0.001) or “people in my neighborhood” (*b* = −0.14, *p* = 0.022). Using the combined social trust measure reported in Figure 2C, we also find a significant association between pathogen disgust sensitivity and generalized social trust no matter whether the question wording targets “most people” (*b* = −0.21, *p* < 0.001) or “people in my neighborhood” (*b* = −0.10, *p* < 0.030). As seen in Figure 2B, for the Yamagishi and Yamagishi (1994) General Trust scale, however, we observe no significant association between pathogen disgust sensitivity and trust in the neighborhood condition (*b* = −0.06, *p* = 0.111), only in the “most people” condition (*b* = −0.14, *p* < 0.001)^11^. Overall, on two out of three trust measures in Figure 2, we observe a significant effect of pathogen disgust sensitivity on trust in “people from my neighborhood.” It is also noteworthy that the effects are descriptively smaller for “people in my neighborhood” relative to “most people,” suggesting that there is not a complete overlap between people's associations for these two groups. To explore this further, we proceed to analyze the subjects' responses to the question battery with the nine different specified groups.

In Table 2, we regress trust in people from the nine specific groups on individual differences in pathogen disgust sensitivity and control variables. Entries in Table 2 are unstandardized OLS regression coefficients with robust standard errors in parentheses. All variables are scaled from 0 to 1 except for age, which is measured in years^12^.

The findings in Table 2, Models 1–9, reveal a striking pattern. Consistent with prior research, we find a substantial and statistically significant relationship between individual differences in pathogen disgust sensitivity and trust in specific outgroups, as presented in Models 6–9 in Table 2. Importantly, consistent with the theoretical prediction that avoidance of people in general is a core output of the behavioral immune system, the findings in Table 2, Models 4–5, also show that individual differences in pathogen disgust sensitivity are a significant predictor of trust in people you meet for the first time (*b* = −0.20, *p* < 0.001) and your neighborhood (*b* = −0.08, *p* = 0.023; replicating the significant effects reported in the experiment). Interestingly, we also observe marginally significant associations between pathogen disgust sensitivity and those with whom the respondents have closer ties—that is, people they know personally (*b* = −0.06, *p* = 0.059 (M3))—and even friends (*b* = −0.06, *p* = 0.063 (M2)). There are no effects on trust in family members.

This pattern clearly suggests that it is not the outgroup status of the group that conditions the effect of pathogen disgust on trust in the group; rather, it is the strength of the social ties. Pathogen avoidance motivations generate distrust in and the avoidance of people to whom you are weakly socially connected, independently of whether they are part of your ingroup network or they constitute an outgroup. This supports our theoretical argument about the primary outputs of the behavioral immune system as well as validate our use of the measure of generalized social trust. Trust in “weak social ties” is exactly what this measure is designed to tap into.

## Study 3: increasing external validity

Studies 1 and 2 are based on convenience samples collected through Amazon's Mechanical Turk platform. The aim of Study 3 was therefore to strengthen the case for the argument that individual differences in pathogen avoidance motivations predict levels of generalized social trust by replicating the analysis in a nationally representative sample. In this way, we can ensure that the results have external validity and do not hinge on the use of a particular subject group. In addition, this study introduced another control variable emphasized in recent research: individual differences in sociosexual orientation (Tybur et al., 2015a,b).

As part of Study 3, we also collected a number of measures of outgroup prejudice. This allows us to compare in further detail the effects of pathogen disgust sensitivity on generalized social trust and outgroup prejudice and to explore whether generalized social trust to some extent constitutes a pathway between pathogen avoidance motivations and prejudice. Most of the existing social science research on generalized social trust has considered trust a more fundamental trait than political ideology and attitudes, such as those reflected in outgroup prejudice (e.g., Lakoff, 1996). Potentially, this could imply that social trust constitutes a crucial core psychological pathway between pathogen avoidance motivations and negative perceptions of outgroups. That is, some of the effects identified in past research could, in part, be indirect and be facilitated because people low in generalized social trust will also tend to distrust outgroup members (Sniderman et al., 2014). Hence, a secondary aim of Study 3 was to investigate whether generalized social trust constitutes a pathway linking pathogen avoidance motivations to negative perceptions of outgroups.

### Materials and measures

We implemented an online survey in the United States. Using quota-sampling, the sample was collected by the YouGov survey agency to match the population on gender, age (18–74), geography, and education. The survey was completed by 2510 respondents, 54% of whom were female, and the average age was 45 years (*SD* = 15 years). In terms of education, 5% of the respondents had no high school diploma, 28% were high school graduates, 26% had some college, 10% had 2 years of college, 20% had 4 years of college, and 11% were post-graduates. The median family income of the respondents was “$40,000 to $49,000.”

As in Studies 1 and 2, pathogen avoidance motivations were measured using the 7-item pathogen disgust scale from the Three Domain Model of Disgust (Tybur et al., 2009). The items form a reliable scale (α = 0.86), ranging from 0 to 1, higher values indicating stronger pathogen disgust sensitivity (mean = 0.64, *SD* = 0.22). Similarly, we used the same standard single-item as in Study 1 to measure social trust, recoded to range from 0 to 1, higher values indicating higher generalized social trust (mean = 0.45, *SD* = 0.26).

To compare the effects of pathogen disgust sensitivity on generalized social trust with the effects on outgroup prejudice, we utilize four measures linked to negative perceptions and attitudes toward outgroups: (1) self-placement on the liberal–conservative continuum; (2) a measure of social conservative issue preferences; (3) attitudes toward immigration, and (4) attitudes toward homosexuals. Clearly, the latter two measures are the only direct measures of outgroup perceptions. In some research, however, political conservatism—and social conservatism in particular—has been argued to reflect caution against outgroups and, hence, provides an important summary marker of the relevant perceptions (e.g., Jost et al., 2009, p. 325; see also Duckitt, 2010, p. 960; Terrizzi et al., 2013, p. 101). We therefore included these two indicators^13^.

Attitudes toward immigrants were measured with three statements (see Slothuus et al., 2010): “Immigrants who are sentenced to jail for crime should be expelled immediately;” “Foreigners should only be eligible for American citizenship once they have learned to behave like Americans;”^14^ “Immigration poses a serious threat to our national identity.” Respondents reported the extent to which they disagreed or agreed with each statement on a 5-point scale (“Don't know” answers were also recorded and subsequently excluded from the analysis). Answers were summed into a reliable scale (α = 0.74) ranging from 0 to 1, higher values indicating more anti-immigrant attitudes (mean = 0.53, *SD* = 0.30).

Attitudes toward homosexuals were assessed with two statements developed to mirror questions selected from the ANES by Treier and Hillygus (2009): “Gay or lesbian couples, in other words homosexual couples, should be legally permitted to adopt children” (reverse coded) and “Homosexuals should be allowed to serve in the United States Armed Forces” (reverse coded). Respondents indicated on a 7-point scale whether they disagreed or agreed with each statement (“Don't know” answers were also recorded and subsequently excluded from the analysis). Answers were summed into a reliable scale (*r* = 0.66) ranging from 0 to 1, higher values indicating stronger opposition toward homosexuals (mean = 0.30, *SD* = 0.31).

To measure self-placement on the liberal–conservative continuum, the respondents were asked, “[g]enerally speaking, do you consider yourself to be…” Answers were obtained on a 7-point scale with endpoints labeled “Very liberal” and “Very conservative” (mean = 0.47, *SD* = 0.28 on a scale ranging from 0 to 1, higher values indicating a more conservative self-placement).

To assess social conservative positions on issues, we included 9 social issue statements in a Likert format (see Online Appendix A5 “Supplemental measurement details for Study 3,” for full question wordings). The questions were developed to mirror the questions selected from the ANES by Treier and Hillygus (2009) to measure the social attitude dimension (two of the items in the social dimension scale were the same as those used for tapping attitudes toward homosexuals). Answers to the items were summed into a reliable scale ranging from 0 to 1, higher values indicating more conservative issue preferences (α = 0.81, mean = 0.43, *SD* = 0.21).

We include the same control variables as in Studies 1–2, i.e., gender, age, education, family income, race (1 = Caucasian, 0 = racial/ethnic minority groups), and personality as indexed by the Big Five. To measure individual differences in personality as indexed by the Big Five inventory, we rely on the 10-item instrument developed by Mondak et al. (2010) (see the Online Appendix A5. “Supplemental measurement details for Study 3,” for full question wordings). All answers were summed into satisfactorily reliable scales ranging from 0 to 1 (r_Openness_ = 0.49, r_Conscientiousness_ = 0.47, r_Extraversion_ = 0.69, r_Agreeableness_ = 0.64, r_Neuroticism_ = 0.75).

In addition to these variables, we also included individual differences in sociosexual orientation as a control variable in Study 3 (e.g., Gangestad and Simpson, 1990) in order to address a potentially extremely important confound in studies of the effects of behavioral immune sensitivity. Tybur et al. (2015a,b) found that the otherwise well-established effect of pathogen avoidance motivations on social conservatism disappeared when controlling for sociosexual orientation; that is, whether one follows a monogamous or promiscuous sexual strategy (Tybur et al., 2015a). Depending on how one interprets the causal ordering of individual differences in sociosexual orientation and disgust sensitivity, this implies that the relationship between pathogen avoidance motivations and social conservatism might be either spurious (if sociosexual orientation precedes disgust sensitivity) or indirect (if disgust sensitivity precede sociosexual orientation) and, hence, potentially cast doubt on whether pathogen avoidance motivations shape social perceptions at all, including generalized social trust.

To measure sociosexual orientation, we rely on a composite measure of seven questions based on the Sociosexual Orientation Inventory (SOI) (Simpson and Gangestad, 1991; see also Penke and Asendorpf, 2008). Subjects were asked questions such as “How many different partners do you foresee yourself having sex with during the next 5 years?”, and asked to report their agreement with statements such as “Sex without love is okay” (see the Online Appendix, A5. “Supplemental measurement details for Study 3,” for full question wordings and details about the index construction). The items were summed into a reliable scale (α = 0.71). The scale was recoded to range from 0 to 1, higher values indicating a more promiscuous sexual strategy (mean = 0.59, *SD* = 0.08).

### Results

To investigate whether pathogen avoidance motivations predict generalized social trust in the general public, we report our findings from three separate model specifications, shown in Table 3. In Model 1 we regress generalized social trust on pathogen disgust sensitivity and the same socio-demographic factors as in Study 1 (cf. Table 1, Model 2). In Model 2 we add controls for Big Five personality traits as in Study 2 (cf. Table 2). In Model 3 we further add control for sociosexual orientation (SOI). Entries in Table 3 are unstandardized OLS regression coefficients with robust standard errors in parentheses. All variables are coded to range from 0 to 1 except for age, which is measured in years^15^.

**Table 3:** **Individual differences in pathogen disgust sensitivity regulate social trust**.

	**M1**	**M2**	**M3**
Pathogen disgust	−0.16^***^ (0.03)	−0.15^***^ (0.03)	−0.15^***^ (0.03)
Education	0.12^***^ (0.02)	0.11^***^ (0.02)	0.11^***^ (0.02)
Income	0.15^***^ (0.03)	0.15^***^ (0.03)	0.15^***^ (0.03)
Caucasian	−0.01 (0.01)	−0.00 (0.01)	−0.00 (0.01)
Female	−0.02^*^ (0.01)	−0.03^*^ (0.01)	−0.03^*^ (0.01)
Age	−0.00 (0.00)	−0.00 (0.00)	−0.00 (0.00)
Openness		0.04 (0.04)	0.04 (0.04)
Conscientiousness		−0.09^**^ (0.03)	−0.09^**^ (0.03)
Extraversion		0.04 (0.03)	0.04 (0.03)
Agreeableness		0.14^***^ (0.04)	0.14^***^ (0.04)
Neuroticism		−0.08^**^ (0.03)	−0.08^**^ (0.03)
SOI			0.05 (0.08)
Constant	0.48^***^ (0.03)	0.43^***^ (0.05)	0.40^***^ (0.07)
*N*	2099	2099	2099
*R*^2^	0.085	0.103	0.103

*Note. Entries are unstandardized OLS regression coefficients. Robust standard errors in parentheses*.

* *p < 0.05*,

** *p < 0.01*,

*** *p < 0.001*.

In all three models, we consistently find that pathogen disgust sensitivity is a substantial and significant predictor of social trust (all *p*'s < 0.001), with higher levels of pathogen disgust sensitivity associated with lower levels of social trust. Crucially, the association between pathogen disgust sensitivity and social trust holds when including both Big Five personality traits (Model 2) and SOI (Model 3) as control variables. These results match those found in Study 1, indicating that the effect generalizes to the broader public and has external validity. Similarly, the findings suggest that the effect is not just due to the association between pathogen disgust sensitivity and SOI that has been detected in past research.

In examining the effect sizes reported in Table 3 of pathogen disgust sensitivity on generalized social trust, we observe that individual differences in pathogen disgust sensitivity predict levels of social trust approximately to the same extent as the direct effect of education and family income. This pattern of results is similar to those from Studies 1 (Table 1) and 2 (Table A4 in the Online Appendix), and, again, strongly suggests that pathogen disgust sensitivity is an important antecedent for generalized social trust.

This conclusion leads us to the secondary aim of Study 3: to directly compare the effects of pathogen disgust sensitivity on generalized social trust and outgroup prejudice and explore whether generalized social trust to some extent constitutes a pathway between pathogen avoidance motivations and prejudice. To this end, we perform observed variable path analyses estimated using SEM.

**Figure 3:**
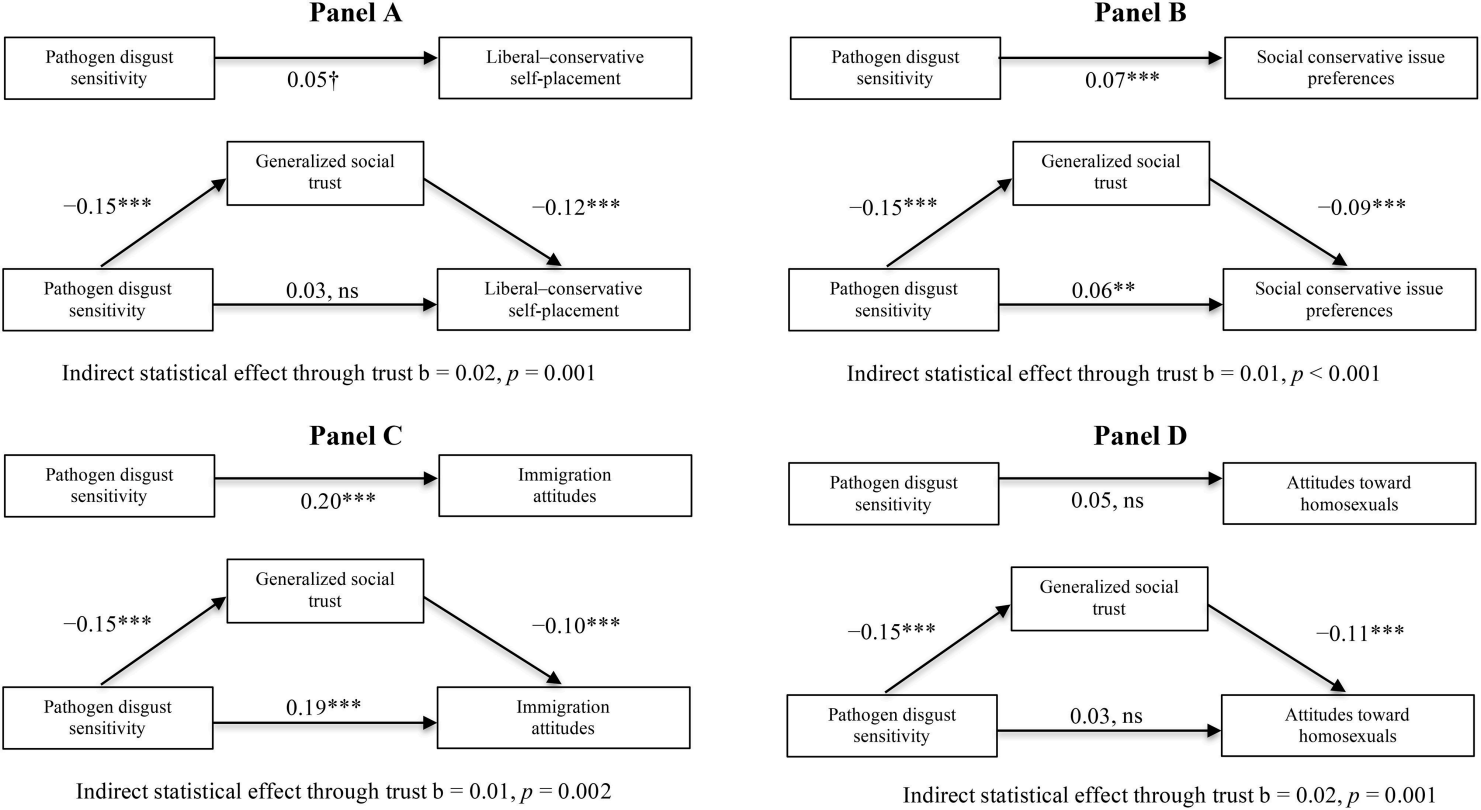
**Generalized social trust as a pathway linking pathogen disgust sensitivity to negative perceptions of outgroups. (A)** Indirect statistical effect through trust *b* = 0.02, *p* = 0.001. **(B)** Indirect statistical effect through trust *b* < 0.01, *p* < 0.001. **(C)** Indirect statistical effect through trust *b* = 0.01, *p* = 0.002. **(D)** Indirect statistical effect through trust *b* = 0.02, *p* = 0.001. Note. In each panel, *n* = 2084. The SEM models were estimated using maximum likelihood estimation. To avoid imposing potentially undue restrictions on the models, we estimate fully saturated models. Gender, age, education, family income, race, Big Five personality traits, and SOI were included as control variables for all effects. The models were estimated using robust standard errors. †*p* = 0.096, ^*^p < 0.05, ^**^p < 0.01, ^***^p < 0.001.

In Figures 3A–D, the upper figure shows the strength of the relationship between pathogen disgust sensitivity and the indicator of outgroup perception without generalized social trust in the model. All of these models include control for gender, age, education, family income, race, Big Five traits, and SOI.

In each panel in Figure 3, the lower figure shows (a) the effect of pathogen disgust sensitivity on generalized social trust, (b) the effect of generalized social trust on the indicator of outgroup perception, and (c) the direct effect of pathogen disgust on the indicator of outgroup perception when including trust in the model. All of the effects are controlled for gender, age, education, family income, race, Big Five personality traits, and SOI.

Finally, in each panel, the line “indirect statistical effect through trust” indicates the indirect effect of pathogen disgust through trust on the indicator of outgroup perception. Entries in Figure 3 are parameter estimates from the path analysis.

The findings in Figure 3 allow us to assess whether generalized social trust significantly accounts for any of the statistical co-variation between individual differences in pathogen disgust sensitivity and outgroup prejudice. Because we are exclusively utilizing cross-sectional data, the analyses cannot tell us anything about the true causal ordering or causal effects of the variables but only about statistical patterns of co-variation.

Reproducing the findings from Table 3, we observe a negative substantial correlation between pathogen disgust sensitivity and generalized social trust across the four panels in Figure 3. Across our three studies (and with several measures included in Study 3), we have thus found replicable effects of pathogen avoidance motivations on generalized social trust, even when controlling for a very large number of potential confounds. When we turn toward the effects of pathogen disgust sensitivity on outgroup prejudice reported in the upper parts of the panels, we see a much less reliable picture. For attitudes toward gays and for liberal–conservative self-placement, there are no discernable direct statistical effects. For social conservative issue preferences, we see a significant but small effect. For attitudes toward immigrants, we see a significant and larger effect that is consistent with the results reported in Study 2. This pattern of wobbly effects on outgroup prejudice suggests again that the link between pathogen avoidance motivations and social cognition is not primarily about facilitating the avoidance of outgroups.

In the lower parts of the panels, we observe a negative correlation between social trust and liberal–conservative ideological self-placement (Figure 3A), social-conservative issue preferences (Figure 3B), immigration attitudes (Figure 3C), and attitudes toward homosexuals (Figure 3D). Together with the reliable effect of pathogen disgust sensitivity on generalized social trust, this suggests that parts of the correlation between pathogen disgust and outgroup prejudice, observed in previous research, could be driven by generalized social trust. Consistent with this, we observe in all four panels that generalized social trust accounts for a small but significant part of the co-variation between individual differences in pathogen disgust sensitivity and negative perceptions of outgroups. As is often the case with indirect statistical effects, the effect sizes are very small. Still, the consistent pattern in the findings across the four indicators suggests that at least parts of the effects of pathogen avoidance motivations on perceptions of outgroups—as identified in past research—are facilitated by the impact of pathogen avoidance motivations on social trust^16^.

It is also important to emphasize that the findings in Figure 3 indicate that for some measures of outgroup perceptions, generalized social trust cannot fully account for the effect of pathogen-avoidance motivations. This is the case for opposition toward immigration but also for general social conservative issue preferences. It is also relevant to note that all models included control for SOI. This suggests that in the case of attitudes toward immigrants and general social conservative issue preferences, SOI cannot fully account for the effects of pathogen disgust as otherwise implied in recent research (Tybur et al., 2015a,b).

## Discussion and conclusions

In this paper, we have tested the prediction that avoidance of people *in general* is a core output of the behavioral immune system. The tests provided supportive evidence, demonstrating a negative correlation between individual differences in pathogen disgust sensitivity and individual differences in generalized social trust. If you tend to worry about pathogens, you will also tend to believe that “most people” should be avoided. Lending credence to the validity of the finding, the correlation was replicated in three studies, including a high-powered, externally valid sample and with the inclusion of a large range of control variables.

Additionally, consistent with our more detailed theoretical argument, the findings in Study 2 supported that individual differences in pathogen disgust sensitivity specifically shape trust in people within an individual's extended social network (which we have referred to as “weak social ties”) and, hence, in people beyond immediate familiarity and beyond outgroups. In Study 2, significant associations between pathogen disgust and trust in people you meet for the first time and people in your neighborhood were observed in addition to the association between pathogen disgust and trust in people from national, religious, and ethnic outgroups. Likewise, the findings from the experiment in Study 2 supported that the effect of pathogen disgust sensitivity on trust was not significantly different when people reported on their trust in people in their neighborhood instead of their trust in “most people.”

We believe that the findings uncovered in this manuscript are important for three reasons. First, they shed important new light on the foundations of individual differences in generalized social trust. In terms of effect sizes, our findings suggest that individual differences in pathogen disgust sensitivity may be at least as important as the resource variables (in particular, income and education) emphasized in past research as the most important individual level factors explaining social trust.

Second, it sheds important new light on the functions of the behavioral immune system. While previous research has argued that the behavioral immune system is designed to specifically tag members of outgroups as pathogenic threats, our analyses show that there is a robust effect on perceptions of “most people.” This suggests that (a) the correlation between general distrust and pathogen avoidance motivations has primacy and (b) the effect on prejudice toward outgroup members might to some extent be a downstream effect of this more general distrust. In Study 3, we provided some supportive evidence for the latter claim.

Third, recent research has called into question whether pathogen avoidance motivations structure social perceptions at all as assessed by individual differences in ideology (Tybur et al., 2015a,b), in part on the basis of skepticism similar to ours about whether outgroups had more dangerous pathogens ancestrally. Instead, Tybur et al. (2015a) argued that correlations between the degree of outgroup prejudice as assessed by the degree of social conservatism and pathogen avoidance motivations could be accounted for by the co-variation between disgust sensitivity and sociosexual orientation. Depending on the causal ordering of pathogen avoidance motivations and sociosexual orientation, this would imply that the effect of pathogen disgust sensitivity on conservatism is spurious or at least indirect. Against the view that pathogen disgust sensitivity does not structure social perceptions, this paper has established a robust empirical relationship between individual differences in pathogen disgust sensitivity and one of the most foundational measures of social perceptions, generalized social trust, even accounting for individual differences in sociosexual orientation and other general personality factors.

In this paper, we have argued that there are solid theoretical reasons to doubt that outgroup members have recurrently carried more dangerous pathogens than ingroup members over human evolutionary history. On this basis, we suggested that the behavioral immune system—in terms of effects on social perceptions—is not foremost a system designed for avoiding outgroup members *per se*. Rather, it is a system designed for avoiding people from one's extended social network and, hence, we argued for the primacy of an effect of disease avoidance motivations on perceptions of people in general.

In closing, however, we want to note that our argument does not necessarily entail that there is no relationship between the group status of an individual and the strength of activation of pathogen avoidance motivations toward that individual. Indeed, in Study 3, we found that pathogen avoidance motivations generate negative perceptions of immigrants over and beyond their effects on generalized trust. There are multiple mechanisms that could account for this. First, previous research has provided evidence that the behavioral immune system operates in a “better safe than sorry” manner such that it tags many non-infectious physical deviations from some prototype (e.g., deformities, disfigurements, or large facial birth marks), as potentially infection risks (e.g., Ryan et al., 2012). Given that ethnic and racial group differences in the modern world often correlate with physical differences (skin color being the most striking) this could imply that outgroup members are tagged as manifestly infected. Second, many cultural practices related to personal hygiene and food preparation are concerned with the regulation of infection risk. Potentially, the behavioral immune system could increase resistance toward the integration of newcomers—without the necessary cultural exposure—into the group as a way to maintain these culturally selected and carefully coordinated practices (Tybur et al., 2015a). Third, pathogen avoidance motivations possibly do not drive outgroup perceptions but *vice versa*. That is, the mechanisms underlying human coalitional psychology are designed to maintain group boundaries for non-pathogen-related reasons (see Kurzban et al., 2001; Tooby and Cosmides, 2010) but could recruit the powerful behavioral immune system to create the required motivation regarding avoidance and opposition. It will be important for future research to disentangle and test whether these psychological dynamics contribute to a link between outgroup avoidance motivation and pathogen avoidance motivations over and above any effects attributable to the fact that those who avoid pathogens will be motivated to avoid people in general independently of their group status.

## Author contributions

LA, MO, and MP all contributed substantially to the research, to the drafting of the work and approved the submitted manuscript. LA, MO, and MP agree to be accountable for all aspects of the research.

## Funding

The research reported in this article has been funded by the Velux Foundation through the grant How to Win with Words (33267).

### Conflict of interest statement

The authors declare that the research was conducted in the absence of any commercial or financial relationships that could be construed as a potential conflict of interest.

## Footnotes

^1^To our knowledge, there are as yet no studies of how the demands for pathogen avoidance (e.g., as measured by the pathogen load of local ecologies) influence parameters such as the extent of social networks in forager groups, the size of bands, the composition of bands (i.e., whether they are mostly composed of kin), and the likelihood of band fusioning. The argument advanced here suggests that such empirical patterns could be relevant to explore.

^2^A potential avenue for achieving protection in the face of within-group variation in pathogen avoidance motivations is the prompt ostracism of those displaying signs of infection. Research on social exclusion among chimpanzees has for example shown that the group rejected chimps who behave abnormally after being infected by polio (Goodall, 1986; see also Kurzban and Leary, 2001, p. 191).

^3^All three authors of the manuscript are employed at the Department of Political Science, Aarhus University, Denmark. The research has been conducted in accordance with institutional and national guidelines. According to these guidelines, ethics approval and written informed consent are not required for survey research in the social sciences. In our study, the participants were invited to participate through either the MTurk platform (Studies 1–2) or YouGov's platform (Study 3) and, hence, we as researchers had no direct access to the participant populations. In the invitation and/or on the first screen in the survey, the participants were briefed about the content of the study before answering any questions. Hence, by proceeding, they consented to participate. The above also applies to Studies 2–3.

^4^Bivariate correlations between pathogen disgust sensitivity and social trust in Study 1 and the subsequent two studies in the paper are reported in Online Appendix A2

^5^We have conducted tests of multicollinearity for all models reported in Table 1. The variance inflation factor (VIF) for each variable in each of the models = 1.15 or less. Hence, the variance inflation factors are satisfactory and multicollinearity is not a problem in the models.

^6^Mean_most people_ = 0.53, SD_most people_ = 0.25, mean_neighborhood_ = 0.59, SD_neighborhood_ = 0.27.

^7^α _most people_ = 0.88, α _neighborhood_ = 0.90, mean_most people_ = 0.62, SD_most people_ = 0.18, mean_neighborhood_ = 0.65, SD_neighborhood_ = 0.18.

^8^Mean_most people_ = 0.58, SD_most people_ = 0.20, mean_neighborhood_ = 0.62, SD_neighborhood_ = 0.21.

^9^As is often the case in interaction models, the VIF scores indicate strong multicollinearity between the interaction term for the effect pathogen disgust sensitivity × experimental condition and the constitutive term for the effect of the experimental condition. Specifically, the VIF for the interaction term for the effect of pathogen disgust sensitivity × experimental condition = 14.26 in all three models and the VIF for the constitutive term for the effect of experimental condition = 13.04 in all three interaction models. Multicollinearity leaves the estimates unbiased but demands high levels of statistical power (see Gujarati, 2003; Sønderskov, 2015). With a sample size of 1420, the models we report should have enough power to detect the existence of interaction effects despite multicollinearity.

^10^Tests for multicollinearity show that the VIF for each variable in each of the six models reported in Figures 2A–C = 1.58 or less. This supports that multicollinearity is not a concern.

^11^A potential concern regarding the construct validity of the Yamagishi and Yamagishi (1994) general trust scale is that items 4 and 5 could seem to capture something slightly different from the other items, just as it is not possible to meaningfully manipulate the target in item 5 “I am trustful” as in the other five items (see Figure 1 above). Importantly, however, item-item correlations for all six items in the Yamagishi and Yamagishi general trust scale are *r* = 0.36 or higher. This support the measurement validity of the scale. Furthermore, analyses of robustness show that the results reported in Figure 2 replicate substantially when items 4 and 5 are excluded from the Yamagishi and Yamagishi (1994) general trust scale. When excluding these items, the effect of pathogen disgust sensitivity on trust becomes marginally significant in the neighborhood condition for the analyses reported in panel b, Figure 2 (b_most people_ = −0.17, *p*_most people_ < 0.001, b_neighborhood_ = −0.08, *p*_neighborhood_ = 0.076) and continues to be significant for the neighborhood condition for the analyses reported in panel c, Figure 2 (b_most people_ = −0.22, *p*_most people_ < 0.001, b_neighborhood_ = −0.11, *p*_neighborhood_ = 0.026). The analyses of robustness also show that the interaction analysis reported in Study 2 replicate when items 4 and 5 are excluded from the Yamagishi and Yamagishi (1994) general trust scale (b_pathogen disgust × experimental condition_ = 0.06, *p* = 0.290 in the model for the Yamagishi and Yamagishi (1994) General Trust scale; and b_pathogen disgust × experimental condition_ = 0.06, *p* = 0.336 in the model for the combined generalized trust measure).

^12^We have conducted tests of multicollinearity for all models reported in Table 2. The VIF for each variable in each of the models = 1.55 or less. This supports that multicollinearity is not a problem in the models.

^13^In this regard, we would like to note that we do not necessarily agree with the narrow conceptualization of political ideology offered in parts of the disgust literature. In our view, political ideology is not only or even primarily related to the regulation of interactions with outgroups. Individual differences in political ideology are also about how to regulate social interactions within the group (see Sniderman et al., 2014, Chapter 4). In this view, political ideology might not be a particularly valid proxy for the degree of negative attitudes toward outgroups.

^14^These items have previously been fielded to respondents in Denmark. We have adapted the second item so that it is meaningful to American respondents by replacing “Danish citizenship” in the original question wording with “American citizenship” and “behave like Danes” with “behave like Americans.”

^15^Tests for multicollinearity for all models reported in Table 3 show that the VIF for each variable in each of the models = 1.36 or less. This indicates that multicollinearity is not a problem in the models.

^16^The findings in Figure 3A are based on self-placement on the liberal–conservative continuum, the most common measure of ideological self-placement in the United States. In the dataset we can also operationalize an alternative measure of ideological self-placement using the respondents self-placement on the left-right scale. Using this alternative measure as the dependent variable and including the same control variables as in Figure 3A, we fail to find a significant indirect effect of pathogen disgust sensitivity on left–right self-placement through social trust (*b* = 0.01, *p* = 0.140, *n* = 2,076). This is due to a non-significant effect of trust on left–right self-placement (*b* = −0.04, *p* = 0.120). Interestingly, this pattern is replicable in the data from Study 1. Here, when differences in statistical power are taken into account, we find an effect of trust on liberal–conservative self-placement (*b* = −0.09, *p* = 0.064) but not on left–right self-placement (*b* = −0.04, *p* = 0.455). The reason why there is a stronger relationship between trust and self-placement on a scale from “liberal” to “conservative” than on a scale from “left” to “right”) is something to address for further research, as it suggests that the two scales tap into different psychological constructs despite their obvious similarity.

## Supplementary material

Replication data and command files are available at Dataverse Network, doi:10.7910/DVN/A6KEW8.
