# Corrigendum: Distrust As a Disease Avoidance Strategy: Individual Differences in Disgust Sensitivity Regulate Generalized Social Trust

**DOI:** 10.3389/fpsyg.2016.01843

**Published:** 2016-12-01

**Authors:** Lene Aarøe, Mathias Osmundsen, Michael Bang Petersen

**Affiliations:** Political Science, Aarhus UniversityAarhus, Denmark

**Keywords:** pathogen avoidance motivation, disgust sensitivity, trust, evolution, outgroup prejudice, generalized social trust, behavioral immune system, ideology

After publication, a very minor coding error was discovered in a single control variable, SOI, used only in Test 3. As consequence, a few of the reported coefficients in Test 3 are slightly different than in the published version. None of the article's conclusions in terms of substance or significance are influenced by this error. The following paragraphs list the corrected coefficients after correction of the coding error:

There is a small mistake in the reported alpha-coefficient, mean, and standard deviation for the SOI index described on page 10 in the article “Distrust As a Disease Avoidance Strategy: Individual Differences in Disgust Sensitivity Regulate Generalized Social Trust” by Aarøe, Osmundsen and Petersen published in Frontiers in Psychology 28 July 2016. The correct values for the SOI index are “α = 0.74” (not 0.71), “*M* = 0.53” (not 0.59), and “*SD* = 0.09” (not 0.08). The same alpha coefficient is reported for the SOI index in the Online Appendix p. 11. On p. 11 in the Online Appendix the correct alpha coefficient should also be “α = 0.74” (not 0.71).

Table 3, Model 3, in the same article contains a small error in the reported unstandardized OLS regression coefficient and standard error for the effect of SOI and for the constant. In Model 3 in Table 3, the correct effect of SOI should be “0.02 (0.07)” [not 0.05 (0.08)] and the correct constant should be “0.42^***^(0.06)” [not 0.40^***^(0.07)].

**Table 3:** **Individual differences in pathogen disgust sensitivity regulate social trust**.

	**M1**	**M2**	**M3**
Pathogen disgust	−0.16^***^ (0.03)	−0.15^***^ (0.03)	−0.15^***^ (0.03)
Education	0.12^***^ (0.02)	0.11^***^ (0.02)	0.11^***^ (0.02)
Income	0.15^***^ (0.03)	0.15^***^ (0.03)	0.15^***^ (0.03)
Caucasian	−0.01 (0.01)	−0.00 (0.01)	−0.00 (0.01)
Female	−0.02^*^ (0.01)	−0.03^*^ (0.01)	−0.03^*^ (0.01)
Age	−0.00 (0.00)	−0.00 (0.00)	−0.00 (0.00)
Openness		0.04 (0.04)	0.04 (0.04)
Conscientiousness		−0.09^**^ (0.03)	−0.09^**^ (0.03)
Extraversion		0.04 (0.03)	0.04 (0.03)
Agreeableness		0.14^***^ (0.04)	0.14^***^ (0.04)
Neuroticism		−0.08^**^ (0.03)	−0.08^**^ (0.03)
SOI			0.02 (0.07)
Constant	0.48^***^ (0.03)	0.43^***^ (0.05)	0.42^***^ (0.06)
*n*	2099	2099	2099
*R*^2^	0.085	0.103	0.103

*Entries are unstandardized OLS regression coefficients. Robust standard errors in parentheses*.

* *p < 0.05*,

** *p < 0.01*,

*** *p < 0.001*.

Figure 3 in the same article contains three small errors: First, in the upper part of Panel C the correct unmediated effect of pathogen disgust sensitivity on immigration attitudes is “0.21^***^” (not 0.20^***^). Second, in the note for Figure 3, the correct text referring to Panel (B) should be “Indirect statistical effect through trust *b* = 0.01, *p* < 0.001” (not “Indirect statistical effect through trust *b* < 0.01, *p* < 0.001). Third, in the note for Figure 3, the alpha level denoted by the “†” symbol should be “^†^*p* = 0.090”, (not ^†^*p* = 0.096).

**Figure 3:**
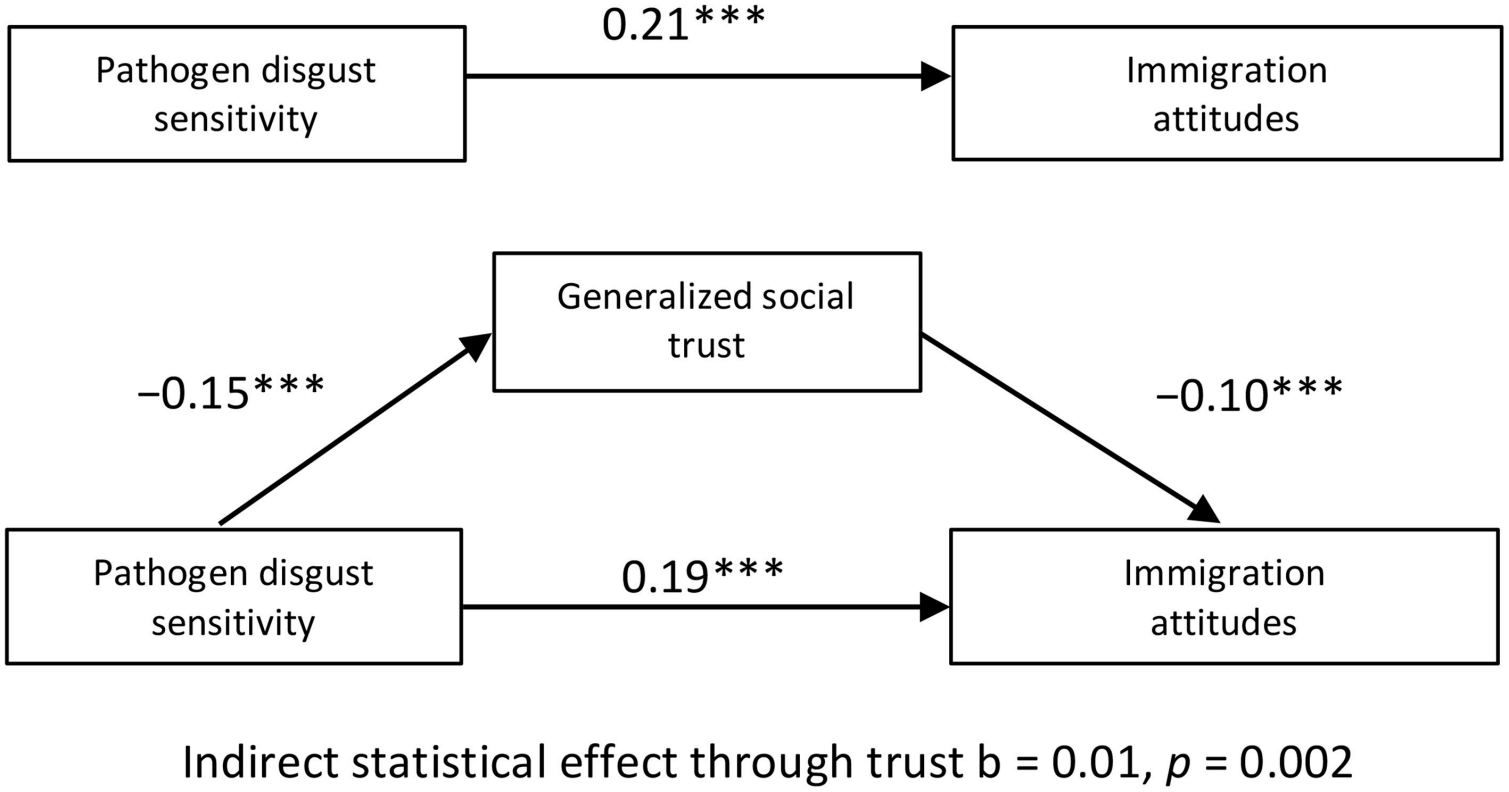
**Panel C**.

Table 3 and Figure 3, Panel C, with corrections appear below in this Corrigendum.

Footnote 16 in the same article contains two small errors in the *p*-values: The *p*-value for the indirect effect of pathogen disgust on left-right self-placement trough social trust should be “*p* = 0.123” (not 0.140) and the *p*-value for the effect of trust on left-right self-placement should be “*p* = 0.102” (not 0.120).

## Ethics statement

All three authors of the manuscript are employed at the Department of Political Science, Aarhus University, Denmark. The research has been conducted in accordance with institutional and national guidelines. According to these guidelines, ethics approval and written informed consent are not required for survey research in the social sciences. In our study the participants were invited to participate through either the MTurk platform (Studies 1-2) or YouGov's platform (study 3) and, hence, we as researchers have no direct access to the participant populations. In the invitation and/or on the first screen in the survey, the participants were briefed about the content of the study before answering any questions. Hence, by proceeding, they were consenting to participate.

## Author contributions

LA, MO and MBP all contributed to the Corrigendum.

## Funding

The research reported in this article has been funded by the Velux Foundation through the grant How to Win with Words (33267).

### Conflict of interest statement

The authors declare that the research was conducted in the absence of any commercial or financial relationships that could be construed as a potential conflict of interest.

