# Supplementary material for: Distrust As a Disease Avoidance Strategy: Individual Differences in Disgust Sensitivity Regulate Generalized Social Trust
Source: Front Psychol. 2016 Jul 28;7:1038. doi: 10.3389/fpsyg.2016.01038 (PMC4963405; doi:10.3389/fpsyg.2016.01038)
Supplement: Supplementary file 1 [file Presentation1.pdf]

Supplemental Online Information for

"Distrust as a Disease Avoidance Strategy: Individual Differences in Disgust Sensitivity Regulate Generalized Social Trust"

Table of Contents

A1. Supplemental measurement details for Study 1 1

A2. Bivariate correlations between pathogen disgust sensitivity and trust measures in Studies 1-3 3

A3. Supplemental measurement details for Study 2 3

A4. Supplemental analysis for Study 2 5
A4.1 Interaction analysis for Study 2 5
A4.2 Regression models for Figure 2 in the main text..6

A5. Supplemental measurement details for Study 3 7

A6. References...11

A1. Supplemental measurement details for Study 1

Education. To measure education, we asked respondents to indicate their last level of completed schooling. They were offered the following response categories: "Less than high school graduate", "High school graduate", "Some college", "Currently a college student", "College graduate", "Post college degree".

For the analysis, the categories "Some college" and "Currently a college student" were collapsed, and the variable was re-scaled to range from 0 to 1, with higher values indicating higher levels of completed schooling (0 = "Less than high school graduate", 0.25 = "High school graduate", 0.50 = "Some college"/"Currently a college student", 0.75 = "College graduate", 1 = "Post college degree").

Income. As our measure of income we rely on family income. Specifically, the respondents were asked to indicate the income category that corresponded to their

family's situation. The following response categories were offered: “Under $15,000”, ”Between $15,000 and $24,999”, ”Between $25,000 and $34,999”, ”Between $35,000 and $49,999”, ”Between $50,000 and $64,999 ”, ”Between $65,000 and $79,999” , ”Between $80,000 and $99,999”, ”Between $100,000 and $149,999”, ”Between $150,000 and $199,999”, ”Over $200,000”.

For the analysis, the variable was re-scaled to range from 0 to 1, with higher values indicating a higher level of income.

Race. To measure race, subjects were asked the following question: “What general racial or ethnic category do you consider yourself...” The following response categories were presented: “Black”, “Caucasian”, “Latino/a or “Hispanic”, “Asian”, “Native American,” and “Other”. For analysis, the variable was coded as a dichotomous measure with 1 = Caucasian and 0 = racial/ethnic minority groups (including ”Black”, “Latino/a or “Hispanic”, “Asian”, “Native American,” and “Other”).

Gender. In the analysis, gender was coded as the dichotomous variable “female” with 1 = female and 0 = male.

A2. Bivariate correlations between pathogen disgust sensitivity and trust measures in Studies 1-3

Table A1 below provides the bivariate correlation between pathogen disgust sensitivity and social trust by study, trust measure, and - in Study 2 – experimental condition.

Table A1: Bivariate correlations between pathogen disgust sensitivity and social trust

	Study 1	Study 2		Study 3
		"most people" condition	"people in my neighborhood" condition	
Single item measure of generalized social trust	-.11*	-.20***	-.14***	-.19***
Yamagishi & Yamagishi general trust scale		-.15***	-.09*	
Combined trust measure		-.20***	-.13***	

Note. Entries are bivariate Pearson's coefficients. * p < 0.05, ** p < 0.01, *** p < 001.

A3. Supplemental measurement details for Study 2

Table A2: Descriptive statistics on the respondents' trust in nine groups

Item	Mean	Standard deviation	Minimum	Maximum	n
"Your family"	0.87	0.21	0	1	1422
"Your friends"	0.80	0.21	0	1	1422
"Your neighborhood"	0.69	0.19	0	1	1422
"People you know personally"	0.55	0.23	0	1	1422
"People you meet for the first time"	0.41	0.24	0	1	1422
"People of another religion"	0.54	0.22	0	1	1422
"People of another nationality"	0.55	0.21	0	1	1422
People of another ethnicity	0.55	0.21	0	1	1422
Immigrants	0.52	0.23	0	1	1422

Note. The respondents were asked "I'd like to ask you how much you trust people from various groups. Could you tell me for each whether you trust people from this group completely, somewhat, not very much or not at all?" Answers were measured on a 4-point scale with endpoints labelled "Do not trust at all" and "Trust completely". All items are coded to range for 0 to 1.

Big Five inventory. To measure individual differences in Big Five traits in Study 2 we rely on the ten-item scale developed by Gosling et al. (2003). Specifically, the participants read the following: “Here are a number of personality traits that may or may not apply to you. For each pair of traits please indicate the extent to which you agree or disagree that the pair of traits applies to you.” ”I see myself as….” ”1. Extraverted, enthusiastic”, ”2. Critical, quarrelsome”, ”3. Dependable, self-disciplined”, ”4. Anxious, easily upset”, ”5. Open to new experiences, complex”, ”6. Reserved, quiet”, ”7. Sympathetic, warm”, ”8. Disorganized, careless”, ”9. Calm, emotionally stable”, ”10. Conventional, uncreative”

Answers were obtained in 7-point scales with endpoints labelled “Disagree strongly” and “Agree strongly”. The items were scored in the following way: Item 1 and item 6 (reverse scored) were combined into the extraversion factor (r = 0.57), item 2 (reverse scored) and item 7 were combined into the agreeableness factor (r = 0.31), item 3 and item 8 (reverse scored) were combined into the conscientiousness factor (0.46), item 4 (reverse scored) and item 9 were combined into the neuroticism factor (r = 0.64) and item 5 and item 10 (reverse scored) were combined into the openness to experience factor (r = 0.35).

Socio-demographic control variables. In Study 2, we measured and coded the socio-demographic variables gender, age, education, family income and race in the same way as in Study 1 (see ”Supplementary measurement details for Study 1” in the Online Appendix for question wording details).

A4. Supplemental analysis for Study 2

A4.1 Interaction analysis for Study 2

The results in Table A3 below show the full interaction models from which the interaction results reported in Study 2 in the main text come.

Table A3: No moderating effect of experimental condition on the impact of pathogen disgust sensitivity on trust (by trust measure).

	Standard single trust item	Yamagishi & Yamagishi (1994) general trust scale	Combined trust measure
	M1	M2	M3
Pathogen disgust	-0.24*** (0.05)	-0.12** (0.04)	-0.18*** (0.04)
"Neighborhood" condition	0.02 (0.05)	-0.00 (0.03)	0.01 (0.04)
"Neighborhood" condition × Pathogen disgust	0.06 (0.08)	0.04 (0.05)	0.05 (0.06)
Education	0.08* (0.03)	0.02 (0.02)	0.05 (0.02)
Income	0.14*** (0.03)	0.11*** (0.02)	0.12*** (0.02)
Caucasian	0.09*** (0.02)	0.06*** (0.01)	0.07*** (0.01)
Female	-0.01 (0.01)	-0.01 (0.01)	-0.01 (0.01)
Age	0.00 (0.00)	0.00 (0.00)	0.00 (0.00)
Openness	-0.06 (0.04)	-0.05 (0.03)	-0.05 (0.03)
Conscientiousness	-0.05 (0.04)	-0.00 (0.03)	-0.03 (0.03)
Extraversion	0.00 (0.03)	0.03 (0.02)	0.02 (0.02)
Agreeableness	0.22*** (0.04)	0.17*** (0.03)	0.20*** (0.03)
Neuroticism	-0.14*** (0.04)	-0.10*** (0.02)	-0.12*** (0.03)
Constant	0.48*** (0.06)	0.54*** (0.04)	0.51*** (0.05)
n	1420	1420	1420
R^2	0.154	0.154	0.172

Note. Entries are unstandardized OLS regression coefficients. Robust standard errors are reported in parentheses. All variables are coded to range from 0 to 1 except for age which is measured in years. $^{*}p < 0.05$, $^{**}p < 0.01$, $^{***}p < 0.001$.

A4.2 Regression models for Figure 2 in the main text

By experimental condition and trust measure the results in Table A4 show the full regression models with the unstandardized OLS regression coefficients for the effects of pathogen disgust sensitivity on trust that are illustrated in Figure 2 in the main text.

Specifically, the results in Panel A in Figure 2 in the main text are based on M1-2 below. The results in Panel B in Figure 2 in the main text are based on M3-4 below. Finally, the results in Panel C in Figure 2 in the main text are based on 5-6 below.

Table A4: The effect of pathogen disgust sensitivity on trust (by trust measure and experimental condition)

	**Standard single trust item**		**Yamagishi & Yamagishi (1994) general trust scale**		**Combined trust measure**	
	**"Some people"**	**"Neigh-borhood"**	**"Some people"**	**"Neigh-borhood"**	**"Some people"**	**"Neigh-borhood"**
	M1	M2	M3	M4	M5	M6
Pathogen disgust	-0.28*** (0.05)	-0.14* (0.06)	-0.14*** (0.04)	-0.06 (0.04)	-0.21*** (0.04)	-0.10* (0.05)
Education	0.06 (0.04)	0.10* (0.04)	-0.01 (0.03)	0.04 (0.03)	0.03 (0.03)	0.07* (0.04)
Income	0.11** (0.04)	0.17*** (0.04)	0.09*** (0.03)	0.12*** (0.03)	0.10*** (0.03)	0.14*** (0.03)
Caucasian	0.03 (0.02)	0.15*** (0.02)	0.02 (0.02)	0.09*** (0.02)	0.02 (0.02)	0.12*** (0.02)
Female	0.03 (0.02)	-0.05* (0.02)	0.01 (0.01)	-0.03* (0.01)	0.02 (0.02)	-0.04* (0.02)
Age	-0.00 (0.00)	0.00 (0.00)	-0.00 (0.00)	0.00 (0.00)	-0.00 (0.00)	0.00 (0.00)
Openness	-0.08 (0.05)	-0.05 (0.05)	-0.05 (0.04)	-0.05 (0.03)	-0.06 (0.04)	-0.05 (0.04)
Conscientious-ness	-0.17** (0.05)	0.06 (0.05)	-0.09* (0.04)	0.09* (0.04)	-0.13** (0.04)	0.08 (0.04)
Extraversion	0.01 (0.04)	0.00 (0.04)	0.01 (0.03)	0.05 (0.03)	0.01 (0.03)	0.02 (0.03)
Agreeableness	0.30*** (0.05)	0.13* (0.06)	0.22*** (0.04)	0.12** (0.04)	0.26*** (0.04)	0.12** (0.04)
Neuroticism	-0.22*** (0.05)	-0.06 (0.05)	-0.14*** (0.04)	-0.06 (0.03)	-0.18*** (0.04)	-0.06 (0.04)
Constant	0.63*** (0.08)	0.35*** (0.08)	0.67*** (0.06)	0.42*** (0.06)	0.65*** (0.07)	0.38*** (0.07)
n	711	709	711	709	711	709
R^2	0.177	0.165	0.147	0.203	0.186	0.198

Note. Entries are unstandardized OLS regression coefficients. Robust standard errors are reported in parentheses. All variables are coded to range from 0 to 1 except for age which is measured in years. $^{*}p < 0.05$, $^{**}p < 0.01$, $^{***}p < 0.001$.

A5. Supplemental measurement details for Study 3

Education. In Study 3 the respondents' educational level was measured using the following categories: 1. "No high school", 2. "High school graduate", 3. "Some college", 4. "2-year", 5. "4-year", and 6. "Post-graduate". For analysis, the variable was re-scaled to range from 0 to 1, with higher values indicating higher levels of education.

Income. As our income measure we rely on family income as in Studies 1-2. Specifically, in Study 3 the respondents' family income was measured using the following response categories: "Less than $10,000", "$10,000 - $19,999", "$20,000 - $29,999", "$30,000 - $39,999", "$40,000 - $49,999", "$50,000 - $59,999", "$60,000 - $69,999", "$70,000 - $79,999", "$80,000 - $99,999", "$100,000 - $119,999", "$120,000 - $149,999", "$150,000 - $199,999", "$200,000 - $249,999", "$250,000 - $349,999", "$350,000 - $499,999", "$500,000 or more" and "Prefer not to say".[1]

In the analysis, the response category "Prefer not to say" was excluded, and the variable was re-scaled to range from 0 to 1, with higher values indicating higher levels of income.

Race. Respondents' racial/ethnic category was measured in the following categories: "Caucasian", "black", "hispanic", "asian", "native American", "mixed", "other", and "middle Eastern". For analysis, race was coded as a dichotomized variable (labeled Caucasian) with 1 = Caucasian and 0 = racial/ethnic minority groups (including "black", "hispanic", "asian", "native American", "mixed", "other", "middle Eastern").

[1] In the data from YouGov, two respondents are indicated to place themselves in an income category labelled "$150,000 or more". Because this answer cannot reliably be coded into one of the income intervals in the income scale answers from these two respondents were excluded on the income variable.

Gender. Gender was coded as a dichotomous variable labelled "female" with 1 = female and 0 = male.

Social conservative issue preferences. To measure social conservative issue preferences, we use nine items developed in a Likert format to mirror the questions selected from the ANES by Treier & Hillygus (2009). Reverse coded questions are marked with "rc". "Please indicate how much you agree or disagree with the following statements?"

1. Gay or lesbian couples, in other words homosexual couples, should be legally permitted to adopt children (rc)
2. If a company has a history of discriminating against blacks when making hiring decisions, then they should be required to have an affirmative action program that gives blacks preference in hiring (rc)
3. By law, a woman should always be able to obtain an abortion as a matter of personal choice (rc)
4. Permission from the parents should be required before a teenage girl under the age of 18 can obtain an abortion
5. Late-term abortions, sometimes called partial birth abortions, should be illegal
6. Homosexuals should be allowed to serve in the United States Armed Forces (rc)
7. The federal government should make it a lot easier for people to buy a gun than it is now
8. Persons convicted of murder should receive the death penalty
9. A woman's place is in the home

Answers were obtained on 7-point scales with endpoints labelled "Disagree strongly" and "Agree strongly". Answers were summed to a highly reliable scale ($\alpha = 0.81$), and re-scaled to range from 0 to 1, higher values indicating strong social issue preferences (mean = 0.43, SD = 0.21).

Big-Five Inventory. To measure individual differences in Big Five traits we relied on the 10-item scale developed by Mondak et al (2010). Specifically, the participants read the following instruction: "The following section contains pairs of words. On the scale, please tell us which word best describes you. If a word is particularly good at describing you, click on the scale next to that word. If neither word describes you, click on the scale in between both words. You can click any

where on the scale. The participants then placed themselves on 11-point scales with the following pairs of words at the endpoints: "An intellectual: Not an intellectual", "Philosophical: Unreflective", "Neat: Sloppy", "Hard working: Lazy" "Outgoing: Shy", "Extraverted: Introverted", "Sympathetic: Unsympathetic", "Kind: Unkind", "Relaxed: Tense", "Calm: Nervous". Below, we report the reliability measures for the five scales: $r_{Openness} = 0.49$, $r_{Conscientiousness} = 0.47$, $r_{Extraversion} = 0.69$, $r_{Agreeableness} = 0.64$, $r_{Neuroticism} = 0.75$.

Sociosexual orientation. To measure individual differences in sociosexual orientation, we used the 7 questions from the Sexual Orientation Inventory (Simpson & Gangestad, 1991). The 7 items include questions about sexual attitudes and overt as well as covert behavior. We used the question wording presented by Penke and Asendorpf (2008: 1114) on the basis of the Sexual Orientation Inventory – SOI (Simpson & Gangestad, 1991). Yet, we changed the wording of item 4 slightly to make the question valid also to participants who were not in a relationship at the time of the study: 1) "With how many different partners have you had sex (sexual intercourse) within the past year?" 2) "How many different partners do you foresee yourself having sex with during the next five years? (Please give a specific, realistic estimate)" 3) "With how many partners have you had sex on one and only one occasion?" 4) "How often do you fantasize about having sex with someone other than your current partner? (if you do not have a partner, then imagine how you would react if you were in a relationship)"[2], 5) " Sex without love is okay", 6) "I can imagine myself being comfortable and enjoying "casual" sex with different partners", 7) "I would have to be closely attached to someone (both emotionally and psychologically)

[2] In the question wording presented by Penke & Asendorpf (2008: 1114), this item was worded "How often do you fantasize about having sex with someone other than your current dating partner".

before I could feel comfortable and fully enjoy having sex with him or her (reverse scored).

Items 1-3 were asked as open-ended questions ("prefer not to answer" options were also provided). Consistent with past research (e.g. Simpson & Gangestad 1991: 883; Webster & Bryan, 2007), higher values on the open-ended items were recoded to reduce problems of "low reliability of the values in the right tail of the distribution due to exaggerations, ballpark estimations, and systematic memory biases" (Penke & Asenpdorpf, 2008: 1116). Answers were recoded to vary between "0 partners" to "9+ partners" (item 1), and "0 partners" to "15+ partners" (item 2-3). Specifically, if a respondent indicated a higher number of partners than "9" when answering item 1 or "15" when answering items 2-3, their answer was recoded to the category "9+ partners" (item 1), and the category "15+ partners" (items 2-3), respectively (see Webster and Bryan (2007: 918-9) for a similar use of "9+ partners" and "15+ partners" as the maximum categories in the recoding of items 1-3). When answering items 1-3 a few respondents indicated an interval of sexual partners, e.g. "2-4" or "1-2". For those respondents we coded the higher number in the interval; e.g. "2" if the respondent indicated "1-2". A very few respondents indicated a minimum of partners; e.g. "10 +". For those respondents we coded the minimum number of partners in their answer; e.g. "10" if they indicated "10+".

Answers to item 4 were indicated on an 8-point scale with endpoints labeled "never" and "at least once a day" ("a prefer not to answer" option was also provided). While Simpson & Gangestad (1991) originally used a 9-point scale to measure agreement with items 5-7, we used 7-point scales ranging from "strongly disagree" to "strongly agree" to ensure consistency with the other Likert items in the survey.

Consistent with the procedure of Simpson and Gangestad (1991: 873), answers to items 5-7 were first aggregated into an index of attitudes toward causal, uncommitted sex (α = .76). Then the index and the other four items were standardized using zscore transformations (Simpson & Gangestad, 1991: 873). “Prefer not to answer” answers were excluded. Finally, the index of attitudes toward causal sex and the other four items were summed to a reliable scale (α = .71). The scale was recoded to vary between 0 and 1 with higher values corresponding to a more unrestricted sexual strategy.

A6. References

Gosling, S. D., Rentfrow, P. J., and Swann, W. B. (2003). A very brief measure of the Big-Five personality domains. *J. Res. Pers.* 37 (6), 504–528

Mondak, J. J., Hibbing, M. V., Canache, D., Seligson, M. A. & Anderson, M. R. (2010): Personality and Civic Engagement: An Integrative Framework for the Study of Trait Effects on Political Behavior, *Am. Polit.l Sci. Rev.*, 104: 85-110.

Penke, L., and Asendorpf, J. B. (2008). Beyond global sociosexual orientations: a more differentiated look at sociosexual orientation and its effects on courtship and romantic relationships. *J. Pers. Soc. Psychol.* 95 (5), 1113–1135.

Simpson, J. A., and Gangestad, S. W. (1991). Individual differences in sociosexual orientation: evidence for convergent and discriminant validity. *J. Pers. Soc. Psychol.* 60 (6), 870–883.

Treier, S., and Hillygus, D. S. (2009). The nature of political ideology in the contemporary electorate. *Public Opin. Q.* 73 (4), 679–703.

Yamagishi, T., and Yamagishi, M. (1994). Trust and commitment in the United States and Japan. *Motiv. Emotion* 18 (2), 129–166.

Webster, G. D., & Bryan, A. (2007). Sociosexual attitudes and behaviors: Why two factors are better than one. *Journal of Research in Personality*, *41*(4), 917-922.
